# Bending the patient safety curve: how much can AI help?

**DOI:** 10.1038/s41746-022-00731-5

**Published:** 2023-01-04

**Authors:** David C. Classen, Christopher Longhurst, Eric J. Thomas

**Affiliations:** 1 University of Utah School of Medicine, Division of Clinical Epidemiology, Salt Lake City, UT USA; 2 UC San Diego Health, Departments of Medicine and Pediatrics, San Diego, CA USA; 3 McGovern Medical School at the University of Texas Health Science Center Houston, and the UT Houston-Memorial Hermann Center for Healthcare Quality and Safety, Houston, USA

**Keywords:** Health policy, Biotechnology

## Abstract

This paper reviews the current state of patient safety and the application of artificial intelligence (AI) techniques to patient safety. This paper defines patient safety broadly, not just inpatient care but across the continuum of care, including diagnostic errors, misdiagnosis, adverse events, injuries, and measurement issues. It outlines the major current uses of AI in patient safety and the relative adoption of these techniques in hospitals and health systems. It also outlines some of the limitations of these AI systems and the challenges with evaluation of these systems. Finally, it outlines the importance of developing a proactive agenda for AI in healthcare that includes marked increased funding of research and evaluation in this area.

## Artificial intelligence and patient safety

Healthcare has been through a rapid digitalization process over the last decade with widespread adoption of electronic health records (EHR) and electronic imaging systems that provide the foundation for a broad spectrum of artificial intelligence (AI) approaches^1–3^. Despite this, patient safety challenges, including measurement and prevention of diagnostic errors, medical errors, adverse events, iatrogenic injury, or other preventable harm to a patient, remain unfortunately common^3^. Current patient safety measurement approaches, which are the minimal requirements to improve safety, are still mostly rooted in the pre digital era with voluntary reporting of safety incidents and extensive manual root cause analysis still commonly used, none of which leverage the new digital infrastructure^3^. Many studies have shown these approaches detect <10% of all safety problems and fail to proactively prevent safety problems^2^. Their use persists because institutional patient safety infrastructure is driven in part by regulations, including Medicare requirements, which focus on manual coding of safety problems using administrative data^3^. However, Medicare has just recently announced the movement to new automated EHR-based safety measures for hospital reporting beginning in 2023, which will catalyze the increasing use of Health IT based approaches in patient safety measurement.

At the same time, AI is increasingly adopted in other industries like banking, aviation, finance, and marketing to improve organizational performance with well documented results. Over the last five years, peer-reviewed publications, on AI in healthcare have increased exponentially, but most have been focused on concepts, development, and initial validation, with few outlining specific operational uses, and even fewer with outcomes assessment, with a few notable exceptions^4^. Given this background, we believe it is time to rigorously assess the use of AI in patient safety and to begin setting an agenda for future research, evaluation, and practice in this field.

A recent study evaluated the use of AI predictive analytics in US health systems with important findings^5^. It found that AI predictive analytics was widely used in these health systems, with 64% have a dedicated team or individual responsible for these AI algorithms and that the most common areas of focus were sepsis identification and hospital readmission risk prediction^5^. As well this study found that almost half of these health systems built their own AI predictive algorithms. This study found similar findings to a survey we did with a group of physician leaders in information technology of US hospitals about their use of AI approaches in the management of patient safety. With 30 responses, we found broad adoption of AI in current clinical operations with more than half of healthcare delivery organizations reporting AI solutions currently deployed in operations, and another 30% planning to do so in the next 2 years. The survey also found that almost half of these applications touched on areas of patient safety such as clinical deterioration scoring, sepsis prediction, surgery complication prediction, and readmission prediction. We also found that almost half of these AI applications were home grown by the organizations themselves rather than created by vendors, supporting the findings in the study mentioned above^5^.

The rapidly increasing use of artificial intelligence (AI) in operational clinical settings presents an opportunity for evaluation as there is limited research or funding for such about its efficacy or safety. There has been steady progress in methods and tools needed to manipulate and transform electronic clinical data, and increasingly mature data resources have supported the rapid development of sophisticated AI in some health care domains including patient safety^6–8^. The broad adoption of personal devices such as wrist watches that measure heart rhythms or portable glucose monitors or other patient self-monitoring devices offer far broader data types than traditional EHR data, and integration of this multi-modal data into EHRs seems likely to yield earlier and more actionable AI predictions^7^. A recent study demonstrated the impact of clinical deterioration software on patient outcomes, this was homegrown in one large health system and has not been disseminated beyond this system. Indeed, there are a large number of vendors currently providing clinical deterioration software products, most of which has not been rigorously evaluated^4^.

There are few rigorous assessments of actual AI deployments in health care delivery systems, and while there is some limited evidence for improved safety processes or outcomes when these AI tools are deployed^4–8^, there is also evidence that these systems can increase risk if the algorithms are tuned to give overly confident results^9^. For example, within AI risk prediction models, the sizeable literature on model development and validation is in stark contrast to the scant data describing successful clinical deployment and impact of those models in health care settings. One study revealed significant problems with one vendor’s EHR sepsis prediction algorithm, which has been very widely deployed among many health systems without any rigorous evaluation^10^.

The prediction of sepsis for inpatients, a common condition with a high mortality rate, is an area of intense AI focus in health care^10–12^. Many studies have shown early detection and treatment of patients with sepsis can markedly reduce mortality. Indeed, a recent review found over 1800 published studies of AI programs developed to predict sepsis in patients hospitalized or in the emergency room. However, none of these models have been widely adopted^11^. The resulting vacuum has been filled by a large commercial EHR vendor that developed its own proprietary model which it deployed to hundreds of US hospitals without any published critical evaluation^10^. One of the health systems that uses this commercial EHR sepsis prediction program performed an evaluation of this program in its own health system. The results were unexpected: the EHR vendor predictive program only picked up 7% of 2552 patients with sepsis who were not treated with antibiotics in a timely fashion and failed to identify 1709 patients with sepsis that the hospital did identify^10^. Obviously, this AI sepsis prediction algorithm was not subjected to rigorous external evaluation but nevertheless was broadly adopted because the EHR vendor implemented it in its EHR package and thus made it conveniently available for its large install base of hospitals^10^. No published evaluation on the impact of this proprietary EHR AI program on patients beyond this hospital has emerged and the impacts both positive and negative that it may have caused in its broad hospital use is unknown.

Another area of opportunity for AI in patient safety is automated interpretation of radiology imaging, which is one of the largest categories of healthcare AI publications over the last 5 years^7^. One example occurred at a large academic health system that had substantial AI resources and a widely used commercial EHR system. Within 2 weeks of the coronavirus pandemic declaration, this health system had already developed, tested, and implemented an AI algorithm for identification of COVID pneumonia on radiology imaging, and found this AI algorithm was both well received and frequently used with a definitive impact on clinical decision making^13^. However, there are other reports that suggest that automated AI imaging diagnostic systems may not be as successful^14^.

These two use cases illustrate important current challenges with the current broad adoption of AI programs across health systems. The sepsis algorithm developed by the EHR vendor came from a large database this vendor has aggregated from client EHR data and used data from 405,000 hospital admissions to develop the algorithm. The fact that the algorithm performed so poorly emphasizes that bigger is not always better and that AI bias, framing, and other issues can exist with AI applications developed even from very large data sets^9,12^. This experience validates many of the current concerns about AI algorithms and outlines the great importance of validating these algorithms in local data sets before they are adopted. The diagnostic Xray example reveals just how quickly these AI algorithms can be developed and deployed within a local institution and perhaps helps to explain why there has been such broad adoption of AI diagnostic imaging algorithms^13^. Clearly the challenges with successful implementation of these AI algorithms reflects in many ways the hurdles and experiences previously identified in deploying EHR based clinical decision support systems^15^. While some impediments were technical, more relate to the complexity of tailoring applications for integration with existing capabilities in electronic health records (EHRs), such as poor understanding of users’ needs and expectations for information, poorly defined clinical processes, workflows, and objectives, and even concerns about legal liability. These impediments may be balanced by the potential for gain, as several reviews of closed malpractice claims found that more than one-half of malpractice claims could have been potentially prevented by well-designed clinical decision support with advanced analytics and AI^16^.

These challenges with the use of AI in healthcare were explored by a conference in October 2021 on patient safety and AI sponsored by the Robert Wood Johnson Foundation (RWJF) where 110 patient safety and Health IT leaders invited from hospital and health systems, health insurers, vendors, researchers, regulators and other stakeholders shared their extensive experiences and approaches to AI and began to build an agenda for patient safety and AI^17^. The attendees described broad adoption of AI in their organizations—despite few published scientific studies of its effectiveness or inherent safety by their organizations. This multistakeholder conference using Delphi like techniques developed a top list of areas that need focused evaluation in AI and Patient Safety, (Table 1) as well as use cases for specific clinical problems in patient safety that are already being used and need further evaluation (Table 2).

**Table 1:** Areas of focus for AI and patient safety per RWJF conference.

1.	Develop AI/advanced analytics implementation models, implementation approaches, and methods for integration into clinical workflows
2.	Create a patient safety framework to guide measurement of AI impact: How to use AI to improve each dimension of safety from retrospective analysis to real-time monitoring to future use of prediction
3.	Build an AI patient safety financial business case
4.	Reduce cognitive and total work burden with AI which should be interpretable and usable for frontline users.
5.	AI patient and consumer focused issues: study how patients and consumers will view and use these tools and how their use will impact patient-doctor and patient-healthcare team relationships
6.	Create ways to engage all the relevant stakeholders in AI use and design
7.	Develop effective governance/oversight and accountability for AI in clinical care
8.	Develop methods to learn and loop back to adjust AI algorithms to ensure equity—refine or change for different or changing populations
9.	Create AI to enhance adverse event/near miss monitoring and real time safety surveillance
10.	Create Use Cases for the application of AI to specific problems in patient safety

**Table 2:** Top use cases for the application of AI to specific clinical problems in patient safety.

1.	Actionable real time patient safety electronic clinical quality measures
2.	Surgical complication prediction
3.	Pressure ulcer prediction
4.	Hypoglycemia prediction
5.	Sepsis prediction
6.	Suicide prediction
7.	Diabetic eye AI screening
8.	Breast imaging cancer screening
9.	Chest x-ray imaging AI diagnosis
10.	Skin melanoma AI diagnosis
11.	Chest x-ray imaging AI cancer screening
12.	Patient self-managed electronic safety dashboards

AI has significant potential to improve patient safety. However, given the lack of rigorous evaluation of AI in actual current practice, coupled with its surprisingly broad use, we believe the time has come to create a national agenda for a critical evaluation of AI and patient safety. This critical evaluation needs to determine among other things whether the broad current adoption of AI in health systems has actually improved patient safety. This agenda must of course include significant new federal research funding for this rigorous AI evaluation, especially in the area of patient safety, if we are to learn from the already wide deployment of AI in healthcare. The Robert Wood Johnson Conference cited above is just the beginning off that process, which should involve major stakeholders such as hospitals and health systems, public and private payors, outcomes researchers, vendors, regulators, and patient advocates and be convened by a trusted entity such as the National Academy of Medicine.
